# Multiparametric Cardiovascular Magnetic Resonance in Acute Myocarditis: Comparison of 2009 and 2018 Lake Louise Criteria With Endomyocardial Biopsy Confirmation

**DOI:** 10.3389/fcvm.2021.739892

**Published:** 2021-10-12

**Authors:** Shuang Li, Xuejing Duan, Guangxun Feng, Arlene Sirajuddin, Gang Yin, Baiyan Zhuang, Jian He, Jing Xu, Wenjing Yang, Weichun Wu, Xiaoxin Sun, Shihua Zhao, Hongyue Wang, Zhongzhao Teng, Minjie Lu

**Affiliations:** ^1^State Key Laboratory of Cardiovascular Disease, Department of Magnetic Resonance Imaging, National Center for Cardiovascular Diseases, Fuwai Hospital, Chinese Academy of Medical Sciences and Peking Union Medical College, Beijing, China; ^2^State Key Laboratory of Cardiovascular Disease, Department of Pathology, Fuwai Hospital, Beijing, China; ^3^State Key Laboratory of Cardiovascular Disease, Department of Cardiology, Fuwai Hospital, Beijing, China; ^4^Department of Health and Human Services, National Heart, Lung and Blood Institute (NHLBI), National Institutes of Health (NIH), Bethesda, MD, United States; ^5^Key Laboratory of Cardiovascular Imaging (Cultivation), Chinese Academy of Medical Sciences, Beijing, China; ^6^State Key Laboratory of Cardiovascular Disease, Department of Echocardiography, Fuwai Hospital, Beijing, China; ^7^State Key Laboratory of Cardiovascular Disease, Department of Nuclear Medicine, Fuwai Hospital, Beijing, China; ^8^Department of Radiology, University of Cambridge, Cambridge, United Kingdom

**Keywords:** myocarditis, cardiovascular magnetic resonance (CMR), diagnostic performance, Lake Louise criteria, endomyocardial biopsy

## Abstract

**Background:** Cardiac magnetic resonance (CMR) has been shown to improve the diagnosis of myocarditis, but no systematic comparison of this technique is currently available. The purpose of this study was to compare the 2009 and 2018 Lake Louise Criteria (LLC) for the diagnosis of acute myocarditis using 3.0 T MRI with endomyocardial biopsy (EMB) as a reference and to provide the cutoff values for multiparametric CMR techniques.

**Methods:** A total of 73 patients (32 ± 14 years, 71.2% men) with clinically suspected myocarditis undergoing EMB and CMR with 3.0 T were enrolled in the study. Patients were divided into two groups according to EMB results (EMB-positive and -negative groups). The CMR protocol consisted of cine-SSFP, T2 STIR, T2 mapping, early and late gadolinium enhancement (EGE, LGE), and pre- and post-contrast T1 mapping. Their potential diagnostic ability was assessed with receiver operating characteristic curves.

**Results:** The myocardial T1 and T2 relaxation times were significantly higher in the EMB-positive group than in the EMB-negative group. Optimal cutoff values were 1,228 ms for T1 relaxation times and 58.5 ms for T2 relaxation times with sensitivities of 86.0 and 83.7% and specificities of 93.3 and 93.3%, respectively. The 2018 LLC had a better diagnostic performance than the 2009 LLC in terms of sensitivity, specificity, positive predictive value, and negative predictive value. T1 mapping + T2 mapping had the largest area under the curve (0.95) compared to other single or combined parameters (2018 LLC: 0.91; 2009 LLC: 0.76; T2 ratio: 0.71; EGEr: 0.67; LGE: 0.73; ). The diagnostic accuracy for the 2018 LLC was the highest (91.8%), followed by T1 mapping (89.0%) and T2 mapping (87.7%).

**Conclusion:** Emerging technologies such as T1/ T2 mapping have significantly improved the diagnostic performance of CMR for the diagnosis of acute myocarditis. The 2018 LLC provided the overall best diagnostic performance in acute myocarditis compared to other single standard CMR parameters or combined parameters. There was no significant gain when 2018LLC is combined with the EGE sequence.

## Introduction

Myocarditis is an inflammatory myocardial disease that is characterized by myocyte necrosis and inflammatory cell infiltrates (1). It is an important cause of cardiac morbidity and mortality that accounts for up to 20% of deaths in adults younger than 40 years (2). About 25% of patients with myocarditis develop persistent cardiac dysfunction, and 12–25% of the patients will rapidly deteriorate or die or progress to dilated cardiomyopathy (3). To timely treat patients, early and correct diagnosis is of the highest importance for patients with myocarditis. However, the diagnosis of myocarditis is challenging due to its diverse etiology and heterogeneous clinical manifestations, going from asymptomatic or subclinical to severe heart failure, arrhythmia, and death.

At present, endomyocardial biopsy (EMB) remains the gold standard for diagnosis of myocarditis (1). However, EMB is an invasive examination, the recommended indications are confined to limited clinical situations, such as life-threatening arrhythmia patients. Cardiac magnetic resonance (CMR) is a noninvasive tool that can characterize myocardial changes for diagnosis of myocarditis (4, 5). In 2009, consensus criteria for CMR in myocardial inflammation known as the Lake Louise Criteria (LLC) were established (6). The myocarditis diagnosis is based on conventional MRI techniques, including T2-weighted imaging (T2WI), early gadolinium enhancement (EGE), and late gadolinium enhancement (LGE), which refer to myocardial edema, hyperemia, and fibrosis, respectively. However, these are qualitative and semi-quantitative diagnostic criteria that mainly depend on visual assessment or signal intensities of a reference tissue sample. For LGE analysis, areas of hyperenhancement were assessed visually. Subtle and diffuse fibrosis were easy to be missed by LGE, as there is no area of normal myocardium with which to compare signal intensity (7). Novel quantitative technology of myocardial T1/T2 mapping can directly measure the T1 and T2 values of myocardial tissue without normal myocardium as a reference. More and more studies have demonstrated the feasibility of mapping technology in the diagnosis of myocarditis (4, 8). Therefore, the LLC were revised in 2018 to recommend that at least one T1-based criterion (T1 mapping, ECV, and LGE) and at least one T2-based criterion (T2WI and T2 mapping) should be used (9). To date, some studies have focused on the diagnostic performance of LLC, but their sample size was relatively small, and they did not use EMB as the gold standard for the diagnosis of myocarditis (10–12). Most of the studies were performed on 1.5T scanner systems, and no comprehensive data for CMR imaging using 3.0 T in patients with acute myocarditis exist so far (11, 13, 14). The study by Luetkens et al. published in 2014 provided the results for a 3.0 T scanner (4), but the diagnostic performance of T2 mapping and the 2018 LLC were not studied. In 2019, the 2018 LLC were added to their investigation (12), but the study mainly validated previously reported cutoff values for parametric mapping techniques and the diagnostic efficiency of different sequences combination was not evaluated. Radunski et al. studied only one combined parameter (LGE + ECV) and did not further analyze any others (14).

The purpose of the present study was to compare the 2009 LLC and the 2018 LLC for the diagnosis of acute myocarditis using 3.0 T MRI with EMB as a reference, further analyzed the diagnostic efficiency of different sequence combinations, and provide the cutoff values for multiparametric CMR techniques.

## Methods

### Subjects

The study was approved by the Research Ethics Committee of the Fuwai Hospital, Beijing, China (approval number: 2020-1274), and all subjects provided written informed consent. Consecutive patients with clinically suspected myocarditis who underwent gadolinium-enhanced CMR and EMB at Fuwai Hospital between January 2016 and December 2020 were enrolled in this study. Myocarditis was suspected in patients who fulfilled the following criteria as recommended by the European Society of Cardiology Working Group on myocardial and pericardial diseases (1): (1) clinical presentations suggestive of myocarditis (acute chest pain, dyspnea at rest or exercise, palpitation, or fatigue); (2) evidence of myocardial damage (functional and structural abnormalities, newly abnormal 12-lead ECG or elevated TnT/TnI). Exclusion criteria included contraindications to CMR, evidence of myocardial infarction, and other cardiac diseases. Patients with infarct patterns of LGE on CMR were also excluded. Cardiac MRI did not influence the diagnostic algorithm. Whether to do biopsy and CMR is decided by the physician according to the patient's condition. As a radiologist, we did not intervene in any examination and treatment of patients. If the physician thinks that the patient needs CMR, we will ask for the patient's consent and scan according to our CMR scanning protocol. Clinical data such as time intervals between the onset of symptoms to cardiac MRI and results of laboratory examination were also collected.

### CMR Protocols and Image Analysis

**Figure 1:**
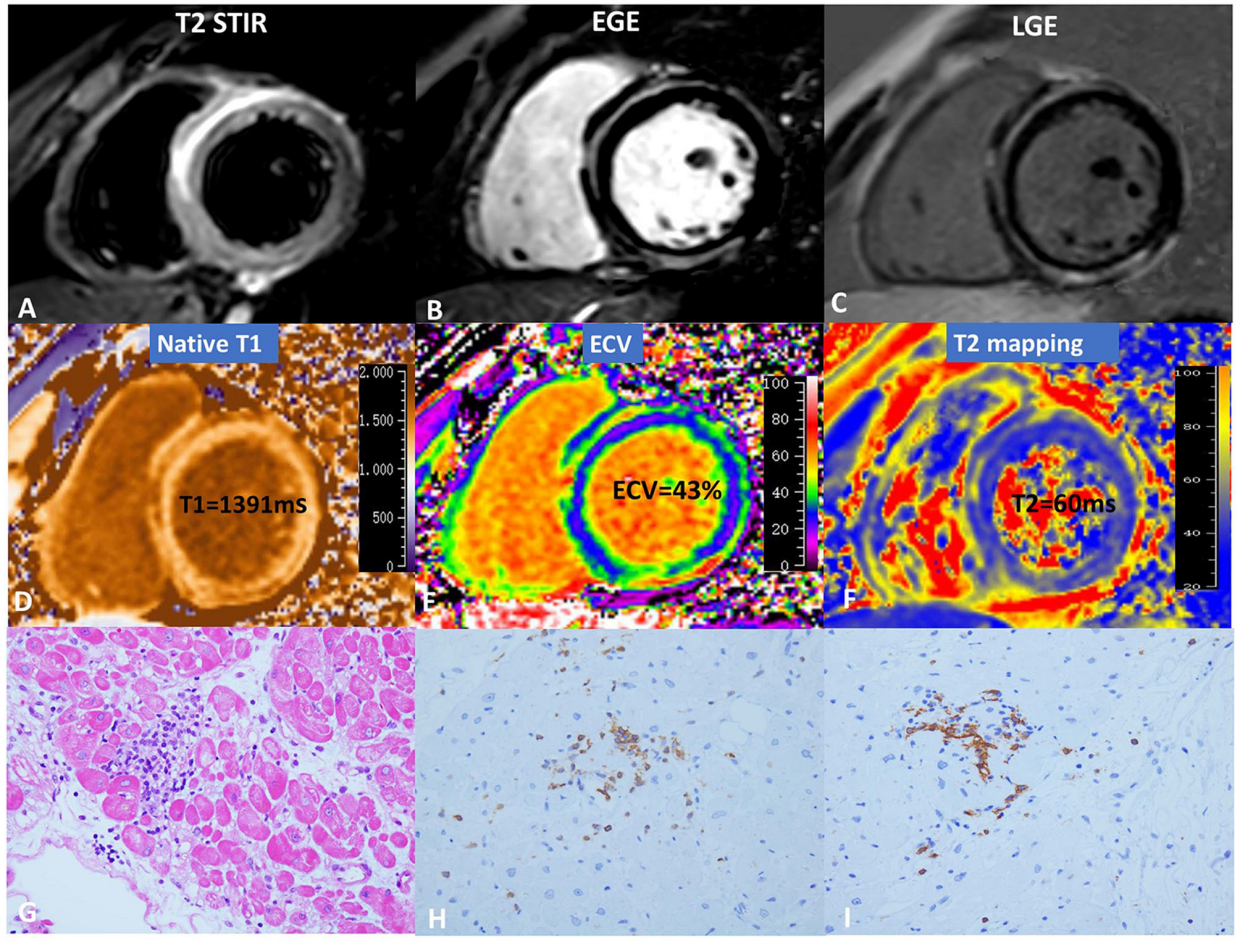
CMR and pathology results for a representative case of myocarditis. **(A)** T2 STIR, **(B)** EGE, **(C)** LGE, **(D)** native T1, **(E)** ECV, and **(F)** T2 mapping. hematoxylin-eosin(HE) staining (×10) **(G)** indicates focal myocyte damage with lymphocytic infiltration. Immunohistochemistry revealed **(H)** LCA + (×40) and **(I)** CD20 + (×40).

Gadolinium-enhanced CMR imaging was performed on a 3.0T scanner (Ingenia; Philips Healthcare, Best, the Netherlands). The balanced steady-state free precession cine images were obtained using short-axis slices from the atrioventricular ring to the left ventricular (LV) apex and two- and four-chamber views with the following parameters: slice thickness: 8 mm, gap: 2 mm, TR: 2.9–3.4 ms, TE: 1.5–1.7 ms, matrix size: 192 × 224–224 × 256, and FOV: 320–380 mm. T2-weighted short tau inversion recovery (T2 STIR) sequences were performed in three LV short-axis (basal, mid, and apical) and four-chamber views (TR: 2 R–R intervals, TE: 80 ms, FOV: 300–380 mm, matrix: 160 × 143, and voxel size: 2.0 × 2.0 × 8 mm). For T1 mapping, a modified Look-Locker inversion recovery (MOLLI) sequence was used for T1 quantification, which was acquired in three LV short-axis views (basal, mid, and apical ventricular short-axis planes). MOLLI images were acquired before intravenous gadolinium administration as well as 15 min after administration of gadolinium (0.2 mmol/kg, gadopentetate dimeglumine, Magnevist^®^, Bayer Healthcare Pharmaceuticals, Wayne, NJ, USA). The pre-contrast MOLLI acquisition followed the 5(3)3 protocol during a breath-hold. Post-contrast MOLLI images followed the 4(1)3(1)2 protocol during a breath-hold. For myocardial T2 mapping, a six-echo gradient spin-echo (GraSE) sequence was used as previously described in the short-axis through basal, mid-ventricular, and apical planes (15). Blood hematocrit levels were determined on the day of examination. EGE was assessed 3 min after intravenous injection of extracellular contrast agent in three LV short-axis views. A spoiled gradient turbo fast low-angle shot sequence with phase sensitive inversion recovery (PSIR) was used for the LGE images, which were acquired 10 min after administration of gadolinium contrast agent using the four-chamber view, two-chamber view, and a series of contiguous 6-mm LV short-axis slices covering the entire LV. Figure 1 shows the CMR and pathology results of a representative case.

All CMR images were transferred to an offline workstation with commercial post-processing software QMass (Medis^®^ QMass, Leiden, the Netherlands) for blinded analysis. Cardiac MRI analysis was performed by a radiologist (S.L. with 4 years of CMR experience) who was blinded to the patients' clinical data and EMB results. The dimensions of the cardiac chambers (left atrium dimension, LAD, LV end-diastolic diameter, and LVEDD), LV volumes (LV end-diastolic volume, LVEDV, LV end-systole volume, and LVESV), and LVEF were measured using standard volumetric techniques (16). LVEDV and LVESV were adjusted for body surface area (BSA) and expressed as indexes. T2 STIR and LGE images were assessed visually and considered positive when a typical pattern of myocarditis was present (6). Semiquantitative T2 signal intensity ratio and EGE ratio were calculated using the signal intensity ratio of the myocardium and the skeletal muscle as recommended (9). LV endocardial and epicardial borders were drawn manually to define the myocardium. Myocardial T1 and T2 values were measured on the basal, mid, and apical short-axis slices according to the American Heart Association 17-segment model (apex excluded), and the global T1 and T2 relaxation times were calculated. Previously described methods were used to calculate the myocardial extracellular volume (ECV) fraction from the T1 map measurements, yielding global ECV fraction values (17, 18).

Inter- and intra-observer variability for T1 and T2 values of the LV segments were assessed in 20 randomly selected subjects, such that one observer performed one measurement, and a second observer blinded to the first observer's results measured two time points at least 1 week apart.

### Endomyocardial Biopsy

EMB was performed with standard techniques as previously described (19). At least three bioptic samples, each 1–2 mm in size, were obtained from the right ventricle in each patient and immediately fixed in 10% buffered formalin at room temperature for light microscopic examination. The principal antibodies used for immunophenotype characterization were CD3, CD20, CD4, CD8, and CD68.

### Statistical Analysis

Statistical analysis was performed using the SPSS 23.0 software. Values are expressed as the mean ± SD or as a percentage, as appropriate. Univariate comparisons were performed using Student's *t*-test, the Mann–Whitney U-test, and Fisher's exact test for normally distributed, non-normally distributed, and categorical variables, respectively. A receiver operating characteristic (ROC) analysis was performed to calculate areas under the curve. The optimal cutoff point was identified using the Youden index, which is the maximum of (sensitivity + specificity-1). Diagnostic accuracy, sensitivities, specificities, accuracies, positive predictive values (PPV), and negative predictive values (NPV) were also calculated. Differences between sensitivities and specificities were calculated with McNemar's test. For the evaluation of intra- and inter-observer variability, the intra-class correlation coefficient was used. Results were considered significant if *p* < 0.05.

## Results

### Population

A total of 73 subjects [aged 32 ± 14 years (14–68 years), 52 males] were enrolled in the present study, 43 of whom were confirmed to have myocarditis by EMB (EMB-positive group). Among 43 patients with EMB confirmed myocarditis, there were 38 lymphocytic myocarditis, three giant cell myocarditis and two eosinophilic myocarditis. Three patients had no immunohistochemical results. Of the remaining 40 patients, 40 patients were CD3 positive, 40 patients were CD4 positive, 39 patients were CD8 positive, 15 patients were CD20 positive, 27 patients were CD68 positive. Of the remaining 30 EMB-negative patients, 18 patients were presenting with arrhythmia, six with dilated cardiomyopathy, one with paraganglioma (bladder), three with pericardial effusion, one with hyperuricosuria, and one with coronary artery anomaly. Most of the patients presenting with arrhythmia were idiopathic, including ventricular tachycardia, sinus bradycardia, ventricular premature contraction and left or right bundle branch block. Only one patient could not be excluded from having arrhythmogenic cardiomyopathy. There were no statistically significant differences in gender or age between the EMB-positive and -negative groups (33 ± 13 years vs. 30 ± 16 years, *p* = 0.50; 67.4 vs.76.7%, *p* = 0.44). The C-reactive protein levels and white blood cell in the EMB-positive group were higher than those in the EMB-negative group (both *p* < 0.05). Thirty-two out of 43 patients in the EMB-positive group had abnormal ECG: three had ventricular fibrillation, 14 had ventricular tachycardia, six had ST-T abnormalities, four had atrioventricular block or bundle branch block, three had sinus arrhythmia, and two had atrial fibrillation. There was no significant difference in time intervals between onset of symptoms to cardiac MRI between the two groups (*p* = 0.93). The baseline characteristics of the study population are detailed in Table 1.

**Table 1:** Clinical characteristics of EMB-positive and EMB-negative groups.

	**All patients (***n*** = 73)**	**EMB positive(***n*** = 43)**	**EMB negative (***n*** = 30)**	***p***
Age (y)	32 ± 14	33 ± 13	30 ± 16	0.500
Male (n)	52 (71.2%)	29 (67.4%)	23 (76.7%)	0.441
BSA (m^2^)	1.78 ± 0.20	1.76 ± 0.19	1.82 ± 0.20	0.259
Heart rate (beats/min)	72 ± 15	75 ± 16	69 ± 12	0.080
HCT (%)	42.0 ± 5.5	42.1 ± 5.1	41.9 ± 6.0	0.870
cTnI (ng/ml)	7.7 ± 7.9	11.3 ± 7.0	1.8 ± 9.4	0.001
WBC (10^3^/μL)	8.3 ± 4.3	9.2 ± 4.9	6.9 ± 3.5	0.019
CRP (mg/L)	15.7 ± 32.3	23.6 ± 40.1	5.0 ± 4.8	0.017
time interval between onset of symptoms to cardiac MRI (d)	10 ± 8	10 ± 8	10 ± 8	0.925
ECG abnormalities (n)	54 (74.0%)	32 (74.4%)	22 (73.3%)	0.917

*BSA, body surface area; HCT, hematocrit; WBC, white blood cell; CRP, C-reactive protein*.

### Diagnostic Performance of Single CMR Parameters

All CMR findings for the two groups are shown in Table 2. There was no significant difference between the two groups in the dimension of the cardiac chambers (LAD: 30.7 ± 10.4 mm vs. 27.7 ± 8.0 mm, *p* = 0.17; LVEDD: 53.8 ± 10.0 mm vs. 50.9 ± 8.0 mm, *p* = 0.17) and cardiac function (48.7 ± 16.9% vs. 50.4 ± 12.3%, *p* = 0.62). T2-ratio (2.3 ± 0.4 vs. 1.7 ± 0.3; *p* = 0.003) and EGEr (4.0 ± 1.0 vs. 3.3 ± 1.2; *p* = 0.014) were significantly higher in the EMB-positive group than in the EMB-negative group. Of the 73 patients, 42 patients (57.5%) had non-ischemic LGE, including 33 patients (76.7%) in the EMB-positive group and nine patients (30.0%) in the EMB-negative group. The myocardial T1 and T2 relaxation times were significantly prolonged in the EMB-positive group compared to the EMB-negative group (1,252 ± 42 ms vs. 1,195 ± 43 ms, *p* < 0.001; 63.2 ± 6.1 ms vs. 54.5 ± 3.7 ms, *p* < 0.001).

**Table 2:** CMR characteristics in EMB-positive and EMB-negative groups.

	**EMB positive (***n*** = 43)**	**EMB negative(***n*** = 30)**	***p***
LAD (mm)	30.7 ± 10.4	27.7 ± 8.0	0.166
LVEDD (mm)	53.8 ± 10.0	50.9 ± 8.0	0.172
LVEF (%)	48.7 ± 16.9	50.4 ± 12.3	0.624
EDVi (mL/m^2^)	95.1 ± 35.3	83.9 ± 29.8	0.150
ESVi (mL/m^2^)	52.3 ± 35.8	43.9 ± 27.2	0.259
T2 ratio	2.3 ± 0.4	1.7 ± 0.3	0.003
EGE ratio	4.0 ± 1.0	3.3 ± 1.2	0.014
LGE	33 (76.7%)	9 (30.0%)	<0.001
Native T1 (ms)	1,252 ± 42	1,195 ± 43	<0.001
ECV (%)	32.7 ± 3.3	29.3 ± 4.1	<0.001
T2 (ms)	63.2 ± 6.1	54.5 ± 3.7	<0.001
2009LLC	34 (79.1%)	8 (26.7%)	<0.001
2018LLC	41 (95.3%)	4 (13.3%)	<0.001

*LAD, left atrium dimension; LVEDD, left ventricular end diastolic diameter; EDVi, index value for left ventricular end-diastolic volume; ESVi, index value for left ventricle end-systole volume; EGE, early gadolinium enhancement ratio; LGE, late gadolinium enhancement; ECV, extracellular volume fraction; LLC, Lake Louise criteria*.

**Figure 2:**
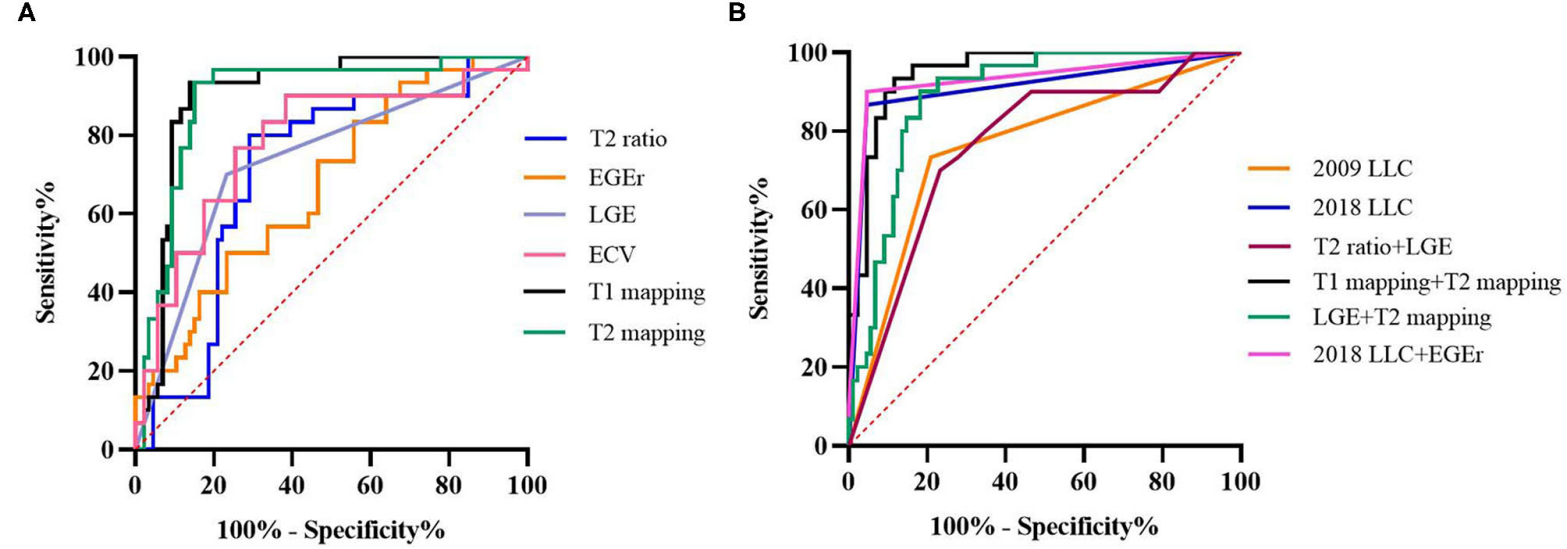
ROC curves for diagnostic performance of CMR parameters and LLC. **(A)** T2 signal intensity (SI) ratio (AUC, 0.71), early gadolinium enhancement ratio (EGEr) (AUC, 0.67), late gadolinium enhancement (LGE) (AUC, 0.73), T1 mapping (AUC, 0.90), extracellular volume (ECV) (AUC, 0.78), and T2 (AUC, 0.90). **(B)** 2009 Lake Louise Criteria (LLC) (AUC, 0.76), 2018 LLC (AUC, 0.91), T2 ratio + LGE (AUC, 0.76), T1 mapping + T2 mapping (AUC, 0.95), 2018 LLC + EGEr (AUC, 0.91), and LGE + T2 mapping (AUC, 0.91).

For the diagnostic efficiency of a single CMR parameter, ROC curves showed that T1 mapping and T2 mapping have the largest area under the curve (AUC) of 0.90 compared to other single CMR parameters (Figure 2A). Optimal cutoff values were 1,228 ms for T1 relaxation times and 58.5 ms for T2 relaxation times, with sensitivities of 86.0 and 83.7%, specificities of 93.3 and 93.3%, positive predictive values of 94.9 and 94.7%, and negative predictive values of 82.4 and 80.0%, respectively. The AUC of EGEr was the smallest (0.76), followed by the T2 ratio (0.71) and LGE (0.73). The diagnostic performance of ECV (AUC-0.78) was second only to T1 and T2 mapping, but it had the lowest sensitivity (65.1%) among all single parameters with a cutoff value of 31.0%. The T2 ratio sensitivity was lower than that of the T2 relaxation times (69.8 vs. 83.7%, *p* = 0.039), although the differences in specificity between the T2 ratio and T2 relaxation times were not statistically significant (80.0 vs. 93.3%, *p* = 0.109). Diagnostic performance and cutoff values for all cardiac MR parameters are provided in Table 3.

**Table 3:** Diagnostic performance of different cardiac MRI parameters for diagnosis of acute myocarditis.

	**Cut off**	**Sensitivity (%)**	**Specificity (%)**	**PPV (%)**	**NPV (%)**	**LR+**	**LR-**	**DOR**
T2 ratio	1.9	69.8	80.0	83.3	64.9	3.5	0.4	8.8
EGEr	3.8	72.1	50.0	67.4	55.6	1.4	0.6	2.3
LGE	–	76.7	70.0	78.6	67.7	2.6	0.3	8.7
Native T1 (ms)	1,228	86.0	93.3	94.9	82.4	12.8	0.2	64.0
ECV (%)	31.0	65.1	83.3	84.8	62.5	3.9	0.4	9.8
T2 (ms)	58.5	83.7	93.3	94.7	80.0	12.5	0.2	62.5
2009LLC	–	79.1	73.3	80.9	71.0	3.0	0.3	10.0
2018LLC	–	95.3	86.7	91.1	92.9	7.2	0.1	72.0

*EGE, early gadolinium enhancement ratio; LGE, late gadolinium enhancement; ECV, extracellular volume fraction; LLC, Lake Louise criteria; PPV, positive predictive value; NPV, negative predictive value; LR+, positive likelihood ratio; LR-, negative likelihood ratio; DOR, diagnostic odds ratio*.

### Diagnostic Performance of LLC and Combined CMR Parameters

**Figure 3:**
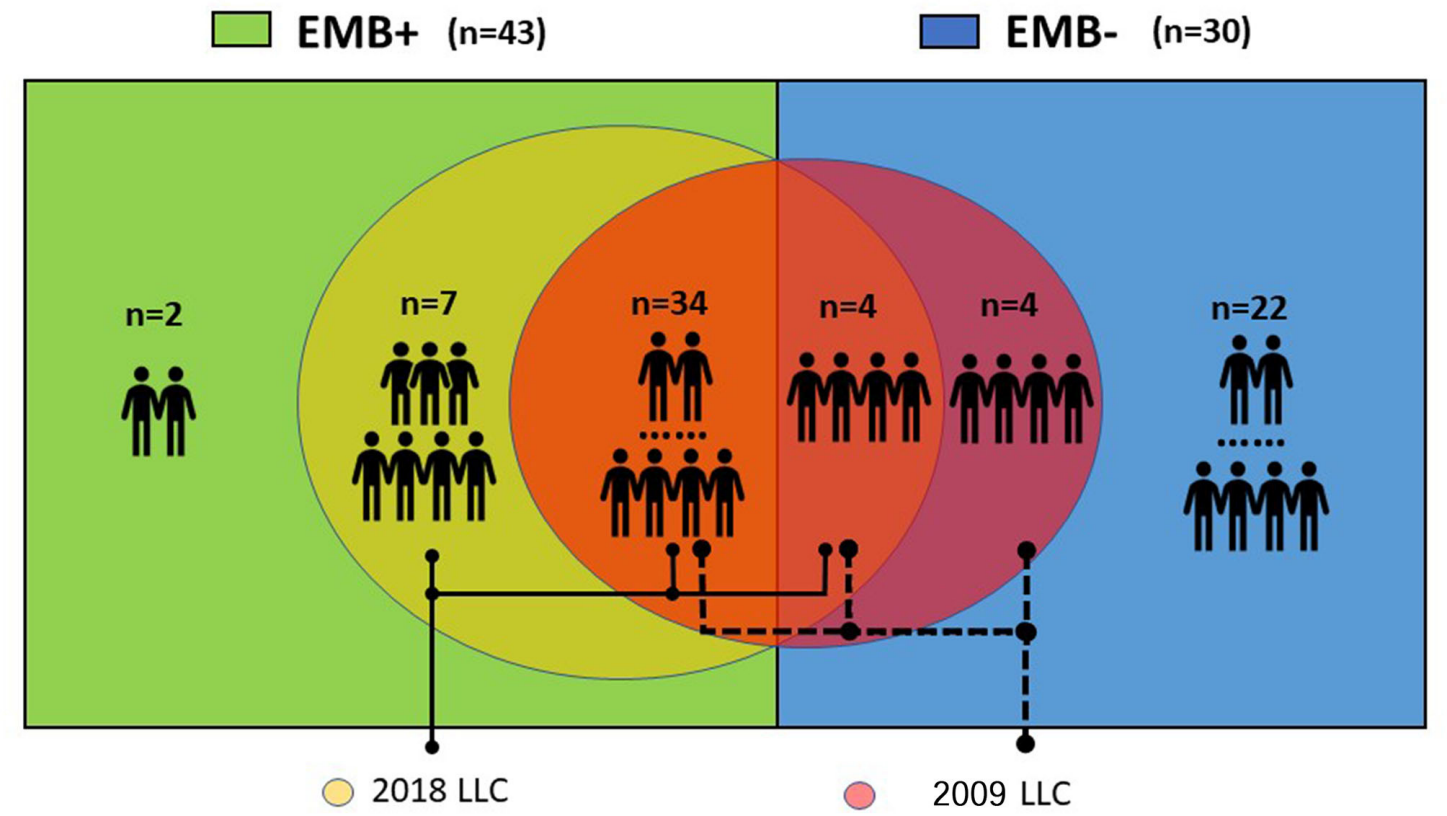
Relationships between 2009 and 2018 LLC with EMB. Eight out of 30 (26.7%) patients without evidence of myocarditis in EMB tests were diagnosed with myocarditis according to the 2009 LLC. All patients diagnosed with myocarditis by the 2009 LLC were correctly identified by the 2018 LLC, but seven patients were missed by the 2009 LLC.

ROC curves showed that the 2018 LLC yielded a higher AUC than the 2009 LLC (0.91 vs. 0.76) with a sensitivity of 95.3% and specificity of 86.7% (Figure 2B). Although the sensitivity and specificity of the 2009 LLC were lower than those of the 2018 LLC, the difference in specificity was not statistically significant (sensitivity: 79.1 vs. 95.3%, *p* = 0.008; specificity: 73.3 vs. 86.7%, *p* = 0.125). All patients diagnosed with myocarditis using the 2009 LLC were correctly identified by the 2018 LLC, although seven patients were missed by the 2009 LLC. Of the 30 patients who had no evidence of myocarditis in the EMB tests, eight patients (26.7%) were diagnosed with myocarditis by 2009 LLC criteria, and four patients (13.3%) were diagnosed with myocarditis by 2018 LLC criteria. The relationships between the 2009 and 2018 LLC with EMB results are shown in Figure 3.

**Figure 4:**
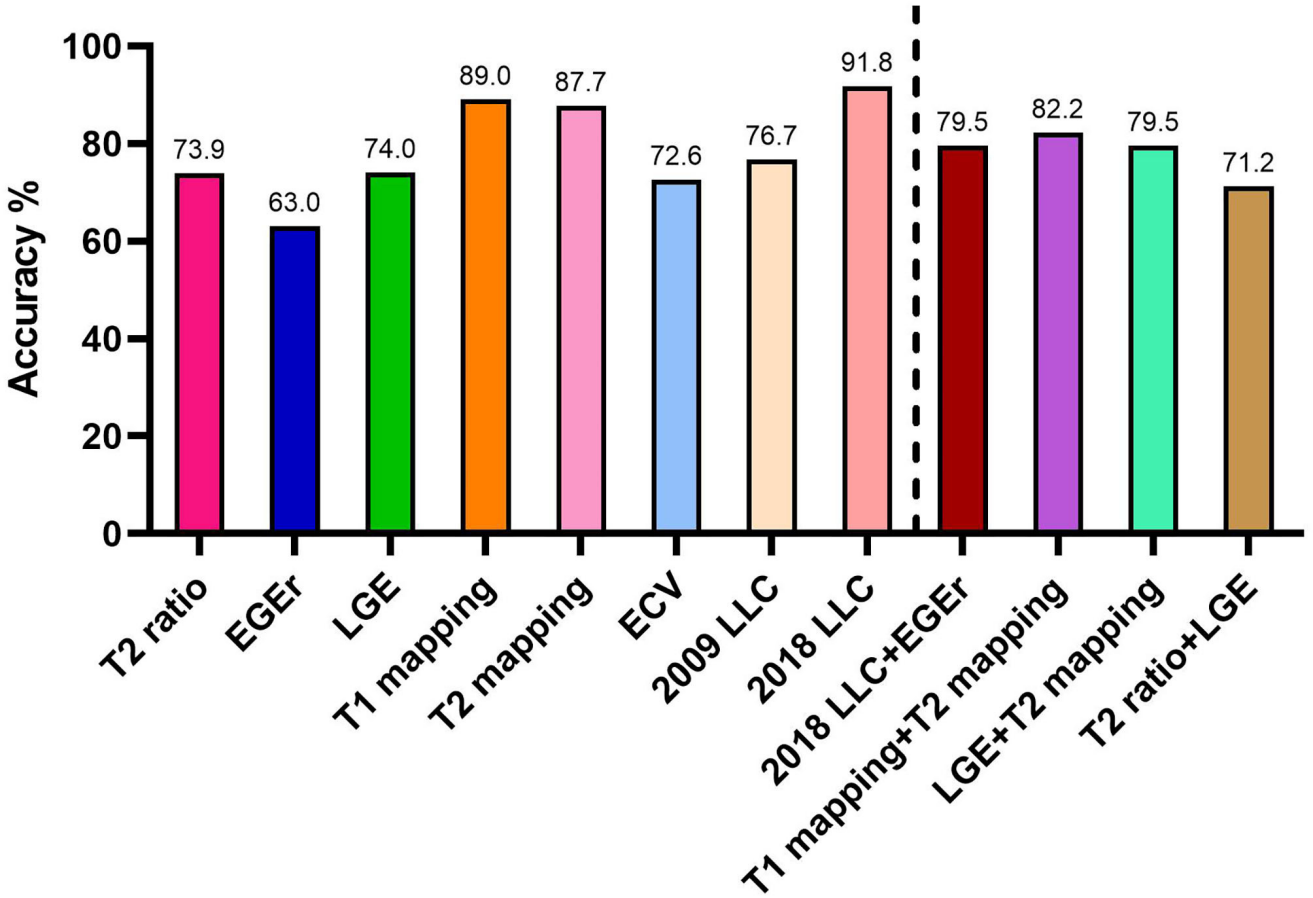
Diagnostic accuracies for CMR parameters and Lake Louise criteria. EGEr, early gadolinium enhancement ratio; LGE, late gadolinium enhancement; ECV, extracellular volume fraction; LLC, Lake Louise criteria.

For the diagnostic performance of combined CMR parameters, the combination of native T1 and T2 mapping showed the best AUC (0.95) among all combined parameters and LLC. When the T2 ratio was combined with LGE, the area under the curve was the smallest (0.76). Although the AUC of T2 ratio + LGE was very similar to 2009 LLC, its diagnostic accuracy was lower (71.2 vs. 76.7%; Figure 4). When the 2018LLC was combined with EGEr, the AUC of 2018LLC + EGEr has little change compared with 2018LLC, but the diagnostic accuracy was significantly reduced (79.5 vs. 91.8%). The combination of LGE and T2 mapping had a higher diagnostic accuracy (79.5%) than the T2 ratio + LGE (71.2%) and a lower diagnostic accuracy than the T1 mapping + T2 mapping (82.2%).

### Intra- and Inter-observer Variability

The intra-class correlation coefficient (ICC) values for inter- and intra-observer variability for T1 relaxation times were 0.96 and 0.97, respectively. The ICC values for inter- and intra-observer variability for T2 relaxation times were 0.95 and 0.96, respectively.

## Discussion

To the best of our knowledge, this is the first study to compare the diagnostic performance between the original 2009 LLC and the updated 2018 LLC with EMB as a reference using 3.0T in a relatively large patient population, further analyzed the diagnostic efficiency of different sequence combinations, and provide the cutoff values for multiparametric CMR techniques. We found that: (1) the 2018 LLC had a better diagnostic performance than the 2009 LLC with respect to sensitivity, specificity, PPV, and NPV, with a cutoff value of 1,228 ms for native T1, 58.5 ms for T2 relaxation times, and 31.0% for ECV; (2) when the 2018 LLC and EGEr were combined, the diagnostic accuracy and precision did not increase compared to the 2018 LLC; and (3) mapping technology significantly improved the diagnostic efficiency. Areas under the curve for T1 mapping + T2 mapping were higher compared to those of other cardiac MR parameters, which is a positive sign for patients who cannot use contrast agents. However, mapping technology is influenced by the field strength and acquisition techniques, thus the reliability of the results still needs to be further evaluated.

### T2WI

Inflammation causes myocardial cell edema, which alters myocardial T2 relaxation time and therefore appears hyperintense on T2WI. Triple inversion recovery turbo spin echo sequences with inversion pulses provide excellent contrast between edema and normal myocardium, improving the detection of myocardial edema (20). But at the same time, the T2WI image quality is affected by many factors, such as sequence parameters, patient's breathing, and arrhythmia. Patients with slow intracardiac blood flow may exhibit an increase in subendocardial blood flow signal, which is easy to misdiagnose (21). This may be the reason why the sensitivity of T2WI in this study was slightly lower (69.8%). The diagnostic accuracy of T2WI was 73.9% in the present study, which was higher than that in the Luetkens et al. study (68%) (4). The cutoff value for the T2 ratio was 2.09 in their study, which was a little higher than the value in the current study (T2 ratio: 1.9).

### EGE

Tissue inflammation causes regional vasodilation, and the increased blood volume in the inflammatory area leads to an increased uptake of contrast agents during the early washout period (6). Therefore, we can assess myocardial hyperemia by calculating the EGEr. However, it had the lowest AUC for diagnosing myocarditis among all of the parameters (0.67), with a specificity of 50.0%. Therefore, it was removed from the LLC in 2018 (9). The present ROC curves showed that the diagnostic precision of T2 ratio + LGE was very similar to that of the 2009 LLC after removing the EGEr from the 2009 LLC. Both of them had an AUC of 0.76. However, the diagnostic accuracy of T2 ratio + LGE was lower than that of the 2009 LLC (71.2 vs. 76.7%). Chu et al. also found that removing the EGE from the 2009 LLC does not significantly reduce diagnostic accuracy for myocarditis, although the positive likelihood ratio may be slightly lower (22). Nevertheless, many experts still recommend using EGE by providers with sufficient experience, as it reflects the tissue changes of the myocardium-hyperemia and capillary leak.

### LGE

Fibrosis caused by severe inflammation can be detected using LGE imaging. Most LGEs occur in the sub-epicardium or mid-myocardium and are more common in the inferolateral and anteroseptal segments, which is very helpful for the diagnosis and differential diagnosis (23). Some studies have shown that LGE has high specificity (80–100%), but poor sensitivity (35–59%) and accuracy (49–71%) (11, 14, 24). The specificity was even lower in our study (70%). This may be because the EMB was used as the gold standard, and the false-negative rate of EMB is high. In our study, nine patients showed a typical LGE enhancement, but there was no evidence of inflammation in the EMB results.

### Mapping/ECV

T1 and T2 relaxation times are obtained on a pixel-by-pixel basis and displayed as maps (9). These novel quantitative techniques can overcome the limitations of conventional LGE in order to assess the diffuse myocardial injury. In the present study, native T1 yielded an excellent diagnostic performance with a sensitivity of 86.0% and a specificity of 93.3%, which is similar to a study by Luetkens et al. (13). At present, the cut-off values for T1 relaxation times are mainly obtained using the 1.5T ranges from 852 to 1,074 ms (4, 13, 25). The cut-off value for T1 mapping was 1,228 ms in the present study. However, there is considerable variability in T1 relaxation times between different field strengths and different acquisition techniques. Therefore, each medical center needs to establish its own normal range for native T1. T2 mapping can detect myocardial water content by quantifying the myocardial tissue T2 relaxation time. Compared to T2WI, T2 mapping was more sensitive to myocardial edema. The sensitivity of T2 mapping was significantly higher than that of the T2 ratio (83.7 vs. 69.8%).

ECV is obtained using T1 maps acquired pre- and post-administration of gadolinium-based contrast agent and adjusted for the hematocrit value. The present study found that ECV had no significant advantage over T1 mapping in the diagnosis of acute myocarditis. It had an AUC of 0.78 with a cutoff value of 31.0%. Our results were similar to those of Luetkens et al. (4). ECV had a sensitivity of 67% and a specificity of 81% in their study. However, a recent study showed that ECV is an independent predictor of adverse cardiovascular events and that T1 mapping could not predict adverse cardiovascular events (26).

### Lake Louise Criteria

The diagnostic performance of the 2018 LLC is better than that of the 2009 LLC in terms of sensitivity, specificity, PPV, and NPV. However, when the 2018 LLC and EGEr were combined, the diagnostic accuracy and precision did not increase compared to the 2018 LLC. The specificity and NPV were significantly decreased by EGEr. Although T1 and T2 relaxation times had the best diagnostic performance for T1WI and T2WI, respectively, the diagnostic accuracy of T1 mapping + T2 mapping was lower than that of the 2018 LLC. The combination of T1 mapping and T2 mapping is a highly attractive option because this protocol is gadolinium-free, which is very useful for patients who cannot use contrast agents.

Although cardiovascular magnetic resonance imaging provides non-invasive tissue characterization of the myocardium and can support the diagnosis of myocarditis, it has little value in identification of infiltrate type, and an attempt to establish cause.

### Limitations

The present study had some limitations. First, this is a single-center study with a relatively small sample size. Second, although EMB was used as the gold standard for diagnosis, it can lead to false-negative results due to sampling errors. Some patients with myocarditis may be included in the EMB negative group. This leads to a decrease in specificity and negative predictive value. Furthermore, EMB was only performed in those subjects with unexplained heart failure and/or new onset arrhythmias which resulted in selection bias. However, the clinical diagnosis is not equal to the real diagnosis of patients, because the specificity of 2013ESC diagnostic criteria is low (27). Third, the dose of contrast agent used in this study is slightly higher. In addition, the results of CMR may have a certain impact on biopsy, but will not cause a great deal of error/bias. As 21(28.8%) of the patients had biopsy before CMR, including 15 CMR positive patients and 6 CMR negative patients in our study cohort.

## Conclusions

The 2018 LLC provided the best overall diagnostic performance in acute myocarditis compared to other single standard CMR parameters or combined parameters. There was no significant gain when 2018LLC is combined with the EGE sequence. Looking for a better combination of CMR sequences and improving the imaging techniques are important directions for future research.

## Data Availability Statement

The raw data supporting the conclusions of this article will be made available by the authors, without undue reservation.

## Ethics Statement

The studies involving human participants were reviewed and approved by the Research Ethics Committee of the Fuwai Hospital, Beijing, China (approval number: 2020-1274). Written informed consent to participate in this study was provided by the participants' legal guardian/next of kin.

## Author Contributions

ML conceived and designed the study. SL wrote the paper. XD and HW performed the pathological analysis. GF enrolled the patients. JX and WY collated the patient's data. BZ and JH did the statistical analysis. GY, WW, and XS scanned the patients. AS and ZT revised the manuscript. ML and SZ guided the whole study. All authors contributed to the article and approved the submitted version.

## Funding

This work was supported by Construction Research Project of Key Laboratory (Cultivation) of Chinese Academy of Medical Sciences (2019PT310025), National Natural Science Foundation of China (81971588 and 81771811), and Capital Clinically Characteristic Applied Research Fund (Z191100006619021), The Capital Health Research and Development of Special (2020-2-4034) and Graduate Innovation Fund of Peking Union Medical College (2019-1002-74).

## Conflict of Interest

The authors declare that the research was conducted in the absence of any commercial or financial relationships that could be construed as a potential conflict of interest.

## Publisher's Note

All claims expressed in this article are solely those of the authors and do not necessarily represent those of their affiliated organizations, or those of the publisher, the editors and the reviewers. Any product that may be evaluated in this article, or claim that may be made by its manufacturer, is not guaranteed or endorsed by the publisher.

## References

[1] Caforio AL Pankuweit S Arbustini E Basso C Gimeno-Blanes J Felix SB. Current state of knowledge on aetiology, diagnosis, management, and therapy of myocarditis: a position statement of the European Society of Cardiology Working Group on Myocardial and Pericardial Diseases. Eur Heart J. (2013) 34:2636–48, 48a-48d. 10.1093/eurheartj/eht210 23824828

[2] Drory Y Turetz Y Hiss Y Lev B Fisman EZ Pines A. Sudden unexpected death in persons less than 40 years of age. Am J Cardiol. (1991) 68:1388–92. 10.1016/0002-9149(91)90251-F 1951130

[3] McCarthy RE III Boehmer JP Hruban RH Hutchins GM Kasper EK Hare JM. Long-term outcome of fulminant myocarditis as compared with acute (nonfulminant) myocarditis. N Engl J Med. (2000) 342:690–5. 10.1056/NEJM200003093421003 10706898

[4] Luetkens JA Doerner J Thomas DK Dabir D Gieseke J Sprinkart AM. Acute myocarditis: multiparametric cardiac MR imaging. Radiology. (2014) 273:383–92. 10.1148/radiol.14132540 24910904

[5] Luetkens JA Schlesinger-Irsch U Kuetting DL Dabir D Homsi R Doerner J. Feature-tracking myocardial strain analysis in acute myocarditis: diagnostic value and association with myocardial oedema. Eur Radiol. (2017) 27:4661–71. 10.1007/s00330-017-4854-4 28500369

[6] Friedrich MG Sechtem U Schulz-Menger J Holmvang G Alakija P Cooper LT. Cardiovascular magnetic resonance in myocarditis: a JACC White Paper. J Am Coll Cardiol. (2009) 53:1475–87. 10.1016/j.jacc.2009.02.007 19389557 PMC2743893

[7] Liang K Baritussio A Palazzuoli A Williams M De Garate E Harries I. Cardiovascular magnetic resonance of myocardial fibrosis, edema, and infiltrates in heart failure. Heart Fail Clin. (2021) 17:77–84. 10.1016/j.hfc.2020.08.013 33220888

[8] Ferreira VM. CMR mapping for myocarditis: coming soon to a center near you. JACC Cardiovasc Imaging. (2018) 11:1591–3. 10.1016/j.jcmg.2018.01.002 29454769

[9] Ferreira VM Schulz-Menger J Holmvang G Kramer CM Carbone I Sechtem U. Cardiovascular magnetic resonance in nonischemic myocardial inflammation: expert recommendations. J Am Coll Cardiol. (2018) 72:3158–76. 10.1016/j.jacc.2018.09.072 30545455

[10] Laissy JP Messin B Varenne O Iung B Karila-Cohen D Schouman-Claeys E. MRI of acute myocarditis: a comprehensive approach based on various imaging sequences. Chest. (2002) 122:1638–48. 10.1378/chest.122.5.1638 12426265

[11] Abdel-Aty H Boye P Zagrosek A Wassmuth R Kumar A Messroghli D. Diagnostic performance of cardiovascular magnetic resonance in patients with suspected acute myocarditis: comparison of different approaches. J Am Coll Cardiol. (2005) 45:1815–22. 10.1016/j.jacc.2004.11.069 15936612

[12] Luetkens JA Faron A Isaak A Dabir D Kuetting D Feisst A. Comparison of original and 2018 Lake Louise criteria for diagnosis of acute myocarditis: results of a validation cohort. Radiol Cardiothorac Imaging. (2019) 1:e190010. 10.1148/ryct.2019190010 33778510 PMC7978026

[13] Luetkens JA Homsi R Sprinkart AM Doerner J Dabir D Kuetting DL. Incremental value of quantitative CMR including parametric mapping for the diagnosis of acute myocarditis. Eur Heart J Cardiovasc Imaging. (2016) 17:154–61. 10.1093/ehjci/jev246 26476398 PMC4882886

[14] Radunski UK Lund GK Stehning C Schnackenburg B Bohnen S Adam G. CMR in patients with severe myocarditis: diagnostic value of quantitative tissue markers including extracellular volume imaging. JACC Cardiovasc Imaging. (2014) 7:667–75. 10.1016/j.jcmg.2014.02.005 24954462

[15] Sprinkart AM Luetkens JA Traber F Doerner J Gieseke J Schnackenburg B. Gradient spin echo (GraSE) imaging for fast myocardial T2 mapping. J Cardiovasc Magn Reson. (2015) 17:12. 10.1186/s12968-015-0127-z 25885268 PMC4326516

[16] Li S Wu B Yin G Song L Jiang Y Huang J. MRI characteristics, prevalence, and outcomes of hypertrophic cardiomyopathy with restrictive phenotype. Radiol Cardiothorac Imaging. (2020) 2:e190158. 10.1148/ryct.2020190158 33778596 PMC7977807

[17] Yang EY Ghosn MG Khan MA Gramze NL Brunner G Nabi F. Myocardial extracellular volume fraction adds prognostic information beyond myocardial replacement fibrosis. Circ Cardiovasc Imaging. (2019) 12:e009535. 10.1161/CIRCIMAGING.119.009535 31838882 PMC7529265

[18] Xu J Zhuang B Sirajuddin A Li S Huang J Yin G. MRI T1 mapping in hypertrophic cardiomyopathy: evaluation in patients without late gadolinium enhancement and hemodynamic obstruction. Radiology. (2020) 294:275–86. 10.1148/radiol.2019190651 31769741 PMC6996717

[19] Leone O Veinot JP Angelini A Baandrup UT Basso C Berry G. 2011 consensus statement on endomyocardial biopsy from the Association for European Cardiovascular Pathology and the Society for Cardiovascular Pathology. Cardiovasc Pathol. (2012) 21:245–74. 10.1016/j.carpath.2011.10.001 22137237

[20] Simonetti OP Kim RJ Fieno DS Hillenbrand HB Wu E Bundy JM. An improved MR imaging technique for the visualization of myocardial infarction. Radiology. (2001) 218:215–23. 10.1148/radiology.218.1.r01ja50215 11152805

[21] Viallon M Mewton N Thuny F Guehring J O'Donnell T Stemmer A. T2-weighted cardiac MR assessment of the myocardial area-at-risk and salvage area in acute reperfused myocardial infarction: comparison of state-of-the-art dark blood and bright blood T2-weighted sequences. J Magn Reson Imaging. (2012) 35:328–39. 10.1002/jmri.22813 21959873

[22] Chu GC Flewitt JA Mikami Y Vermes E Friedrich MG. Assessment of acute myocarditis by cardiovascular MR: diagnostic performance of shortened protocols. Int J Cardiovasc Imaging. (2013) 29:1077–83. 10.1007/s10554-013-0189-7 23404383

[23] Sebai F Brun S Petermann A Ribes D Prevot G Cariou E. Cardiac magnetic resonance imaging with late gadolinium enhancement in acute myocarditis: towards differentiation between immune-mediated and viral-related aetiologies. Arch Cardiovasc Dis. (2019) 112:559–66. 10.1016/j.acvd.2019.09.001 31648948

[24] Voigt A Elgeti T Durmus T Idiz ME Butler C Beling M. Cardiac magnetic resonance imaging in dilated cardiomyopathy in adults–towards identification of myocardial inflammation. Eur Radiol. (2011) 21:925–35. 10.1007/s00330-010-1985-2 20963443

[25] Pan JA Lee YJ Salerno M. Diagnostic performance of extracellular volume, native T1, and T2 mapping versus Lake Louise criteria by cardiac magnetic resonance for detection of acute myocarditis: a meta-analysis. Circ Cardiovasc Imaging. (2018) 11:e007598. 10.1161/CIRCIMAGING.118.007598 30012826 PMC6192699

[26] Grani C Biere L Eichhorn C Kaneko K Agarwal V Aghayev A. Incremental value of extracellular volume assessment by cardiovascular magnetic resonance imaging in risk stratifying patients with suspected myocarditis. Int J Cardiovasc Imaging. (2019) 35:1067–78. 10.1007/s10554-019-01552-6 30756221

[27] Biesbroek PS Hirsch A Zweerink A Van De Ven PM Beek AM Groenink M. Additional diagnostic value of CMR to the european society of cardiology (ESC) position statement criteria in a large clinical population of patients with suspected myocarditis. Eur Heart J Cardiovasc Imaging. (2018) 19:1397–397:i. 10.1093/ehjci/jex308 29186442

